# Supplementary figures and images for: serojump: A Bayesian tool for inferring infection timing and antibody kinetics from longitudinal serological data
Source: medRxiv. 2025 Mar 5:2025.03.04.25323335. Preprint. [Version 1] doi: 10.1101/2025.03.04.25323335 (PMC11908275; doi:10.1101/2025.03.04.25323335)

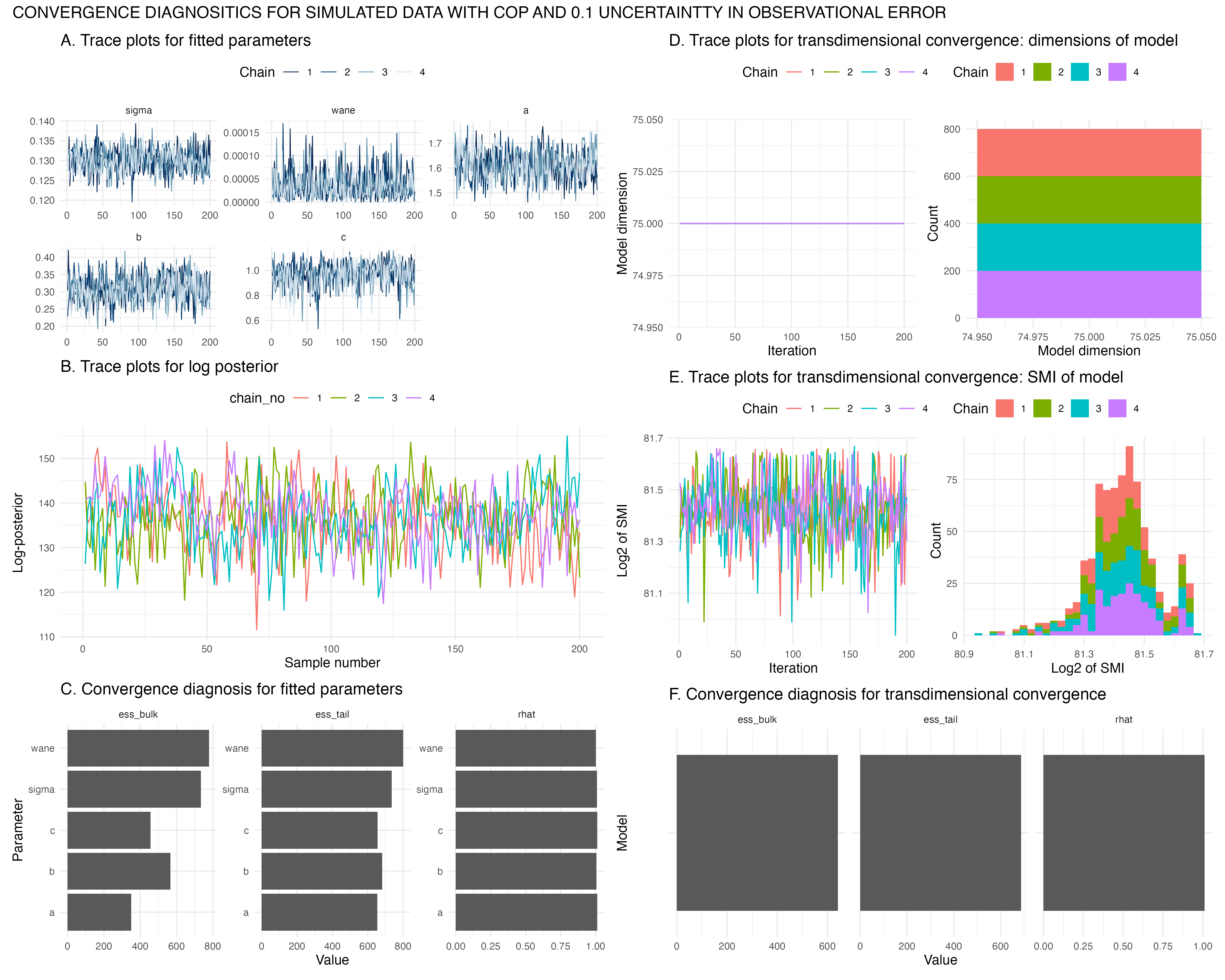

Supplement: Supplement 2 — Figure S1: Convergence diagnostics for simulated data with COP and 0.1 uncertainty in observational error. (A) Trace plots for fitted parameters (sigma, wane, a, b, c) across four Markov chains, illustrating the mixing and convergence of the parameters. (B) Trace plots for the log posterior across the four chains, showing the variability and stabilization of the log posterior over iterations. (C) Convergence diagnostics for fitted parameters, including effective sample size (ess_bulk, ess_tail) and Rhat, which assess the adequacy of sampling and convergence for each parameter. (D) Trace plots for transdimensional convergence of the model dimension, with histogram counts of model dimensions sampled across the chains. (E) Trace plots for transdimensional convergence for the SMI (Structural Model Index) and histogram counts of the log-transformed SMI values across chains. (F) Convergence diagnostics for transdimensional parameters, including effective sample size (ess_bulk, ess_tail) and Rhat, summarizing the adequacy of sampling and convergence for the transdimensional space. [file media-2.jpg]

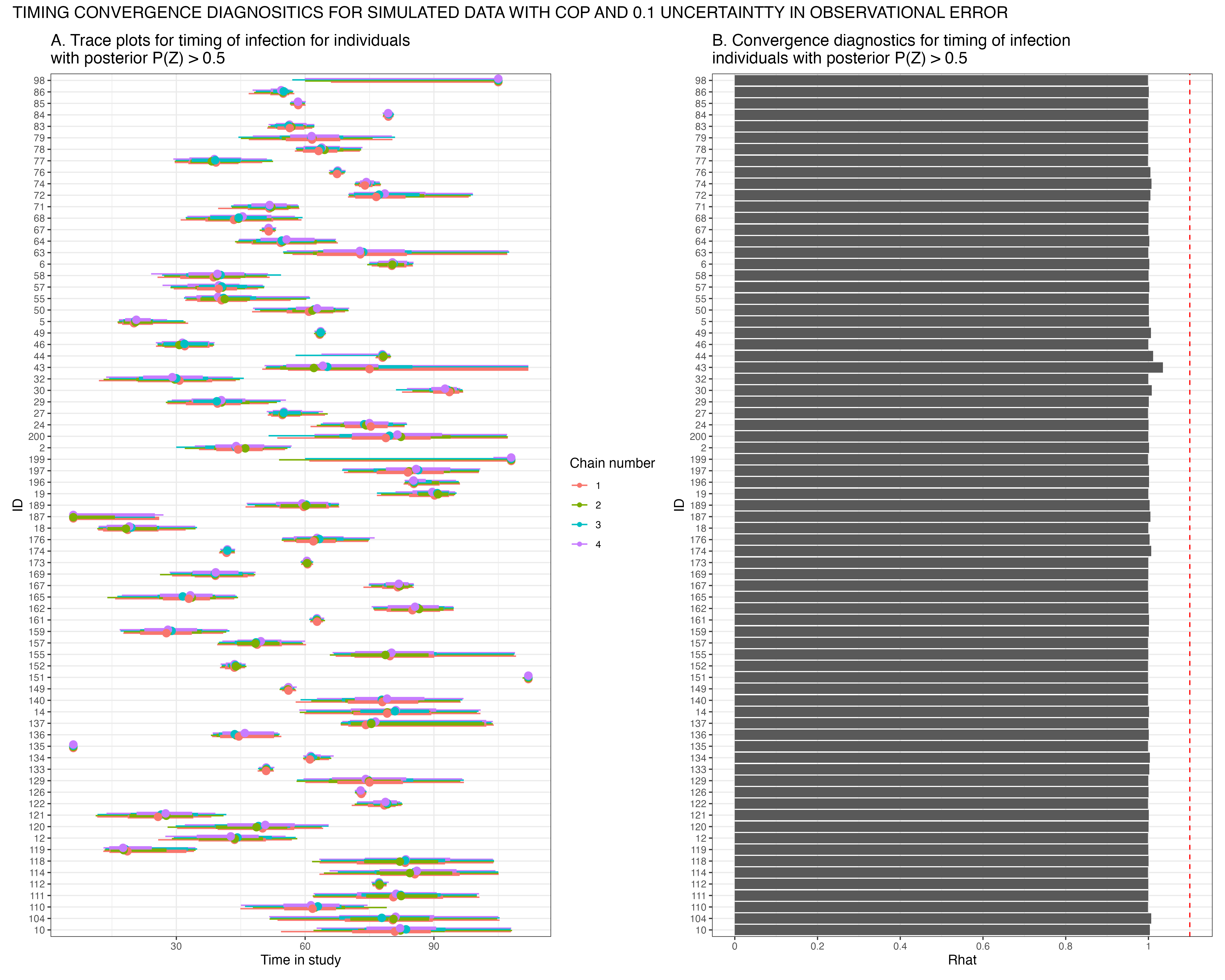

Supplement: Supplement 3 — Figure S2: Convergence diagnostics of infection timing for simulated data with COP and 0.1 uncertainty in observational error. (A) Trace plots for the timing of infection for individuals with posterior P(Z) > 0.5 display estimates across four Markov chains. Each point and its uncertainty interval reflect the sampled infection timing for each individual over iterations. (B) Convergence diagnostics for the timing of infection for individuals with posterior P(Z)>0.5, showing Rhat values for each individual. The red dashed line indicates the threshold for Rhat=1.1, which marks convergence. [file media-3.jpg]

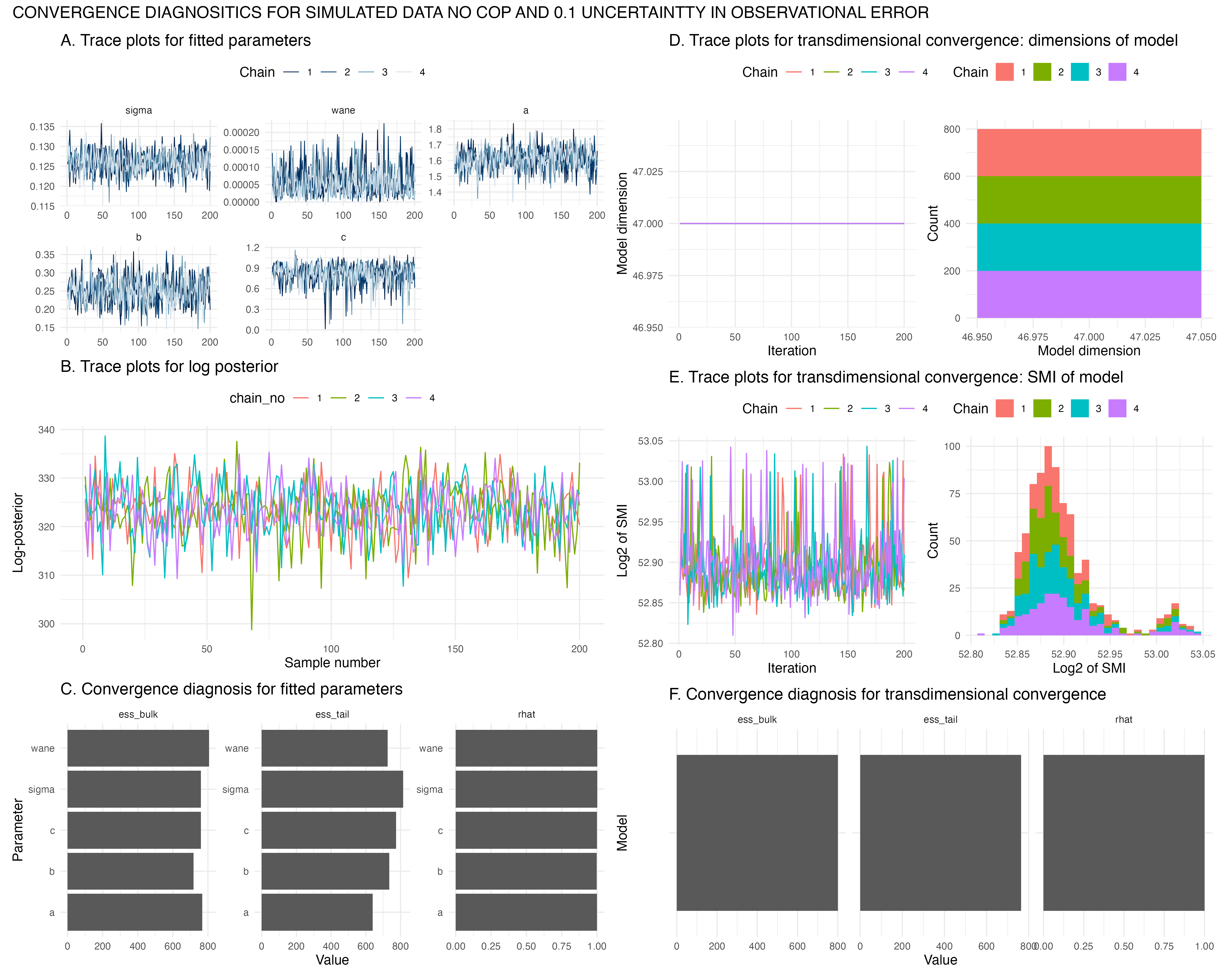

Supplement: Supplement 4 — Figure S3: Convergence diagnostics for simulated data No COP and 0.1 uncertainty in observational error. (A) Trace plots for fitted parameters (sigma, wane, a, b, c) across four Markov chains, illustrating the mixing and convergence of the parameters. (B) Trace plots for the log posterior across the four chains, showing the variability and stabilization of the log posterior over iterations. (C) Convergence diagnostics for fitted parameters, including effective sample size (ess_bulk, ess_tail) and Rhat, which assess the adequacy of sampling and convergence for each parameter. (D) Trace plots for transdimensional convergence of the model dimension, with histogram counts of model dimensions sampled across the chains. (E) Trace plots for transdimensional convergence for the SMI (Structural Model Index) and histogram counts of the log-transformed SMI values across chains. (F) Convergence diagnostics for transdimensional parameters, including effective sample size (ess_bulk, ess_tail) and Rhat, summarizing the adequacy of sampling and convergence for the transdimensional space. [file media-4.jpg]

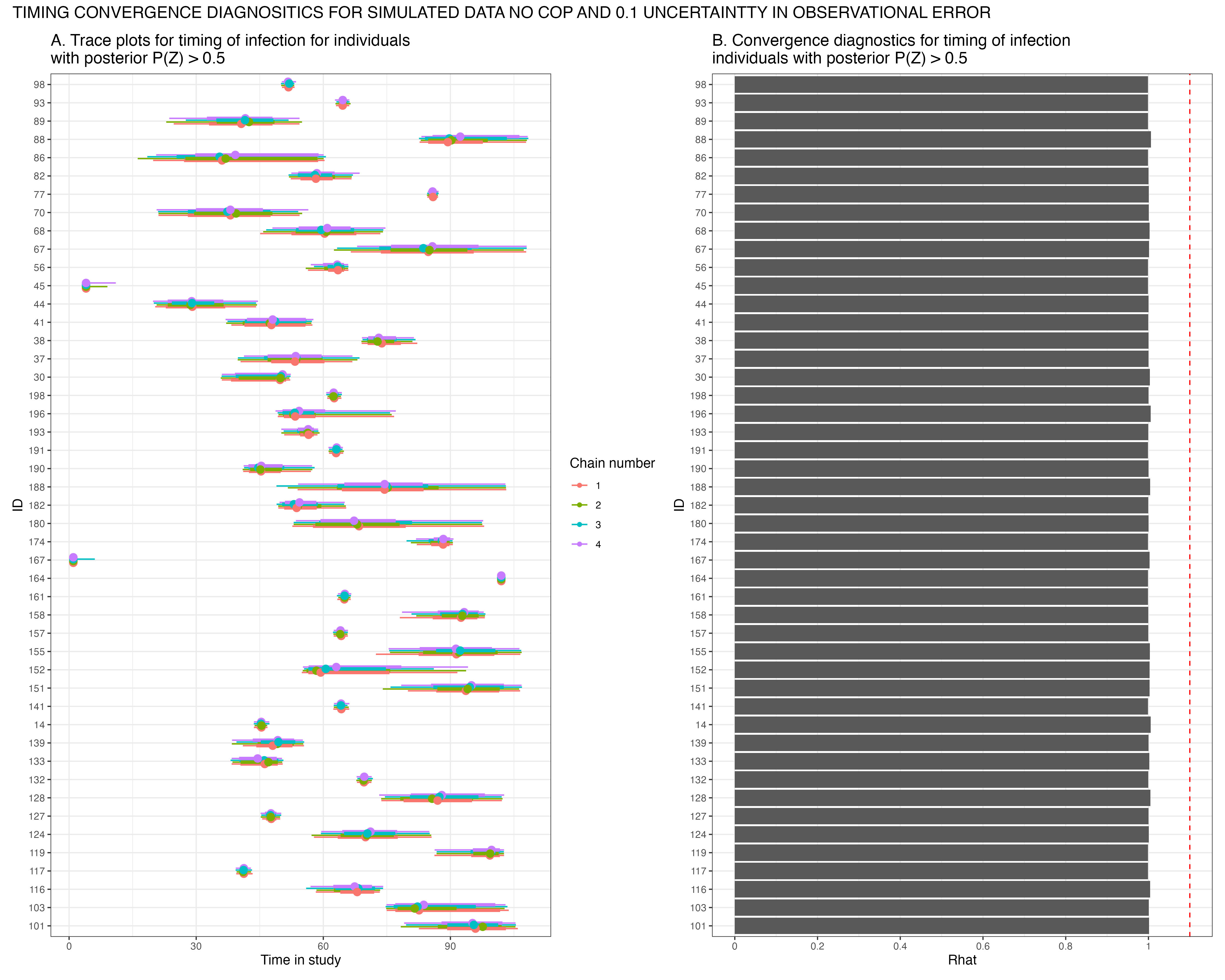

Supplement: Supplement 5 — Figure S4: Convergence diagnostics of infection timing for simulated data No COP and 0.1 uncertainty in observational error. (A) Trace plots for the timing of infection for individuals with posterior P(Z) > 0.5 display estimates across four Markov chains. Each point and its uncertainty interval reflect the sampled infection timing for each individual over iterations. (B) Convergence diagnostics for the timing of infection for individuals with posterior P(Z)>0.5, showing Rhat values for each individual. The red dashed line indicates the threshold for Rhat=1.1, which marks convergence. [file media-5.jpg]

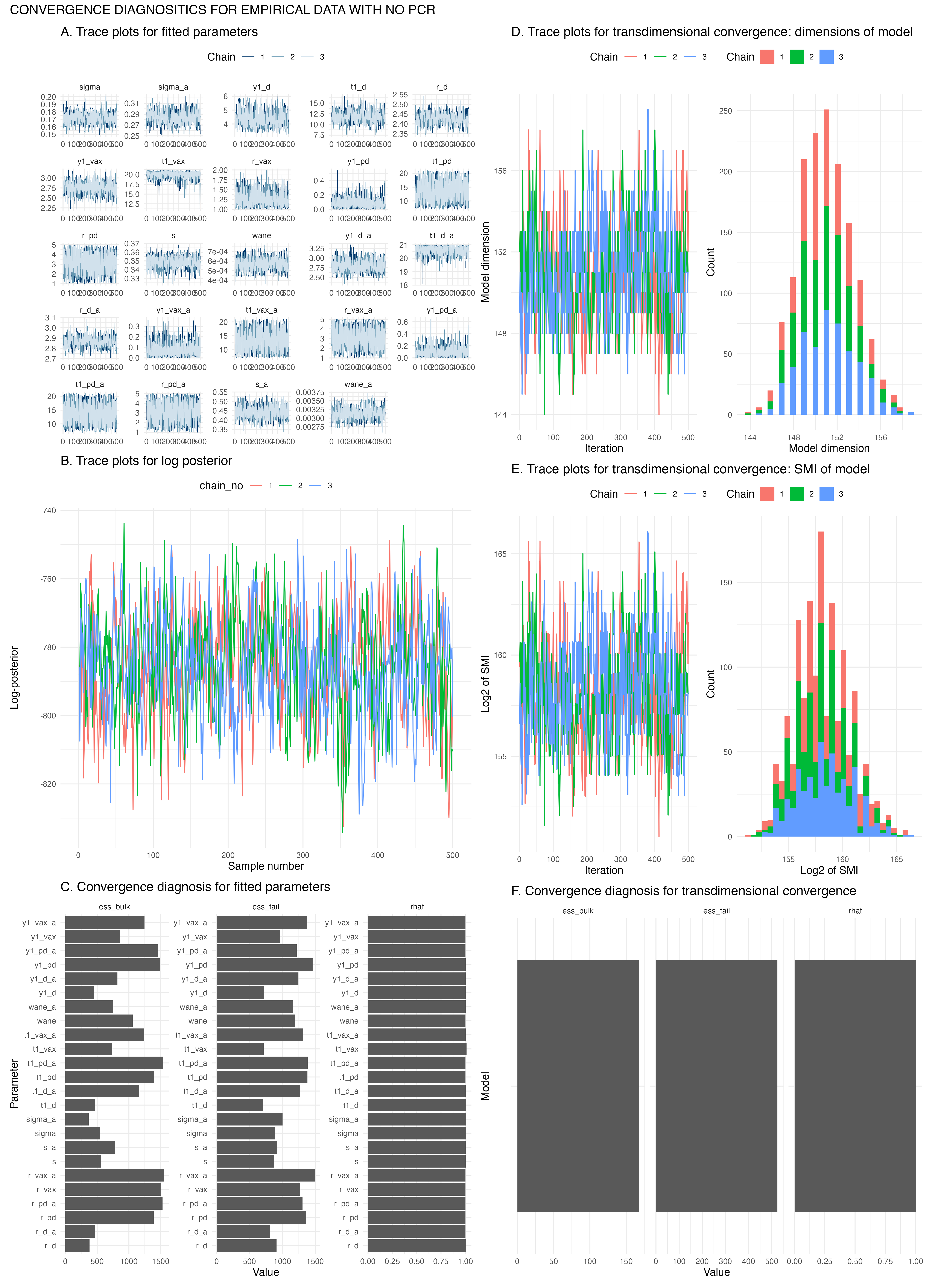

Supplement: Supplement 6 — Figure S5: Convergence diagnostics for empirical data without PCR information (A) Trace plots for fitted parameters (sigma, wane, a, b, c) across four Markov chains, illustrating the mixing and convergence of the parameters. (B) Trace plots for the log posterior across the four chains, showing the variability and stabilization of the log posterior over iterations. (C) Convergence diagnostics for fitted parameters, including effective sample size (ess_bulk, ess_tail) and Rhat, which assess the adequacy of sampling and convergence for each parameter. (D) Trace plots for transdimensional convergence of the model dimension, with histogram counts of model dimensions sampled across the chains. (E) Trace plots for transdimensional convergence for the SMI (Structural Model Index) and histogram counts of the log-transformed SMI values across chains. (F) Convergence diagnostics for transdimensional parameters, including effective sample size (ess_bulk, ess_tail) and Rhat, summarizing the adequacy of sampling and convergence for the transdimensional space. [file media-6.jpg]

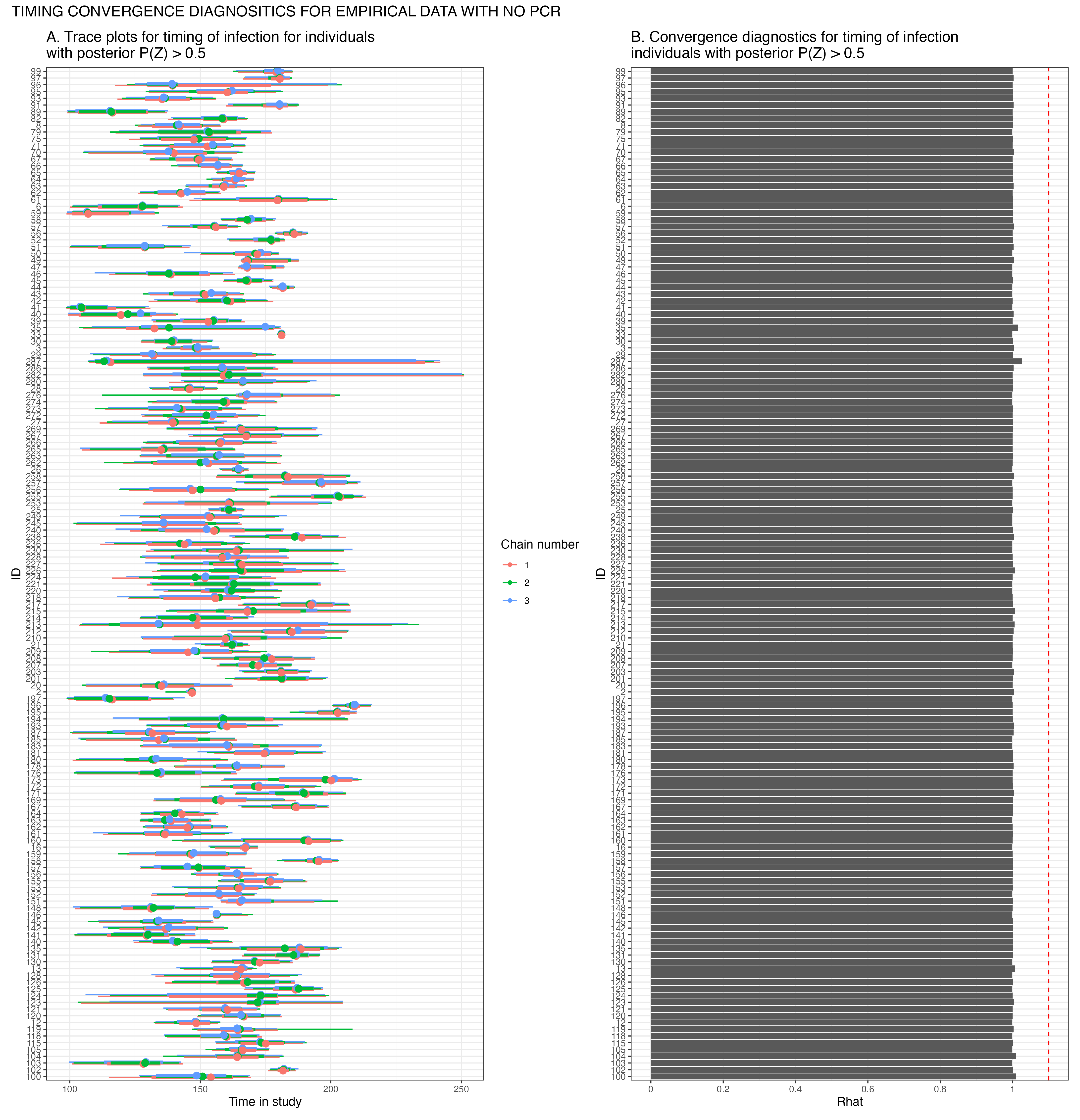

Supplement: Supplement 7 — Figure S6: Convergence diagnostics for empirical data without PCR information. (A) Trace plots for the timing of infection for individuals with posterior P(Z) > 0.5 display estimates across four Markov chains. Each point and its uncertainty interval reflect the sampled infection timing for each individual over iterations. (B) Convergence diagnostics for the timing of infection for individuals with posterior P(Z)>0.5, showing Rhat values for each individual. The red dashed line indicates the threshold for Rhat=1.1, which marks convergence. [file media-7.jpg]

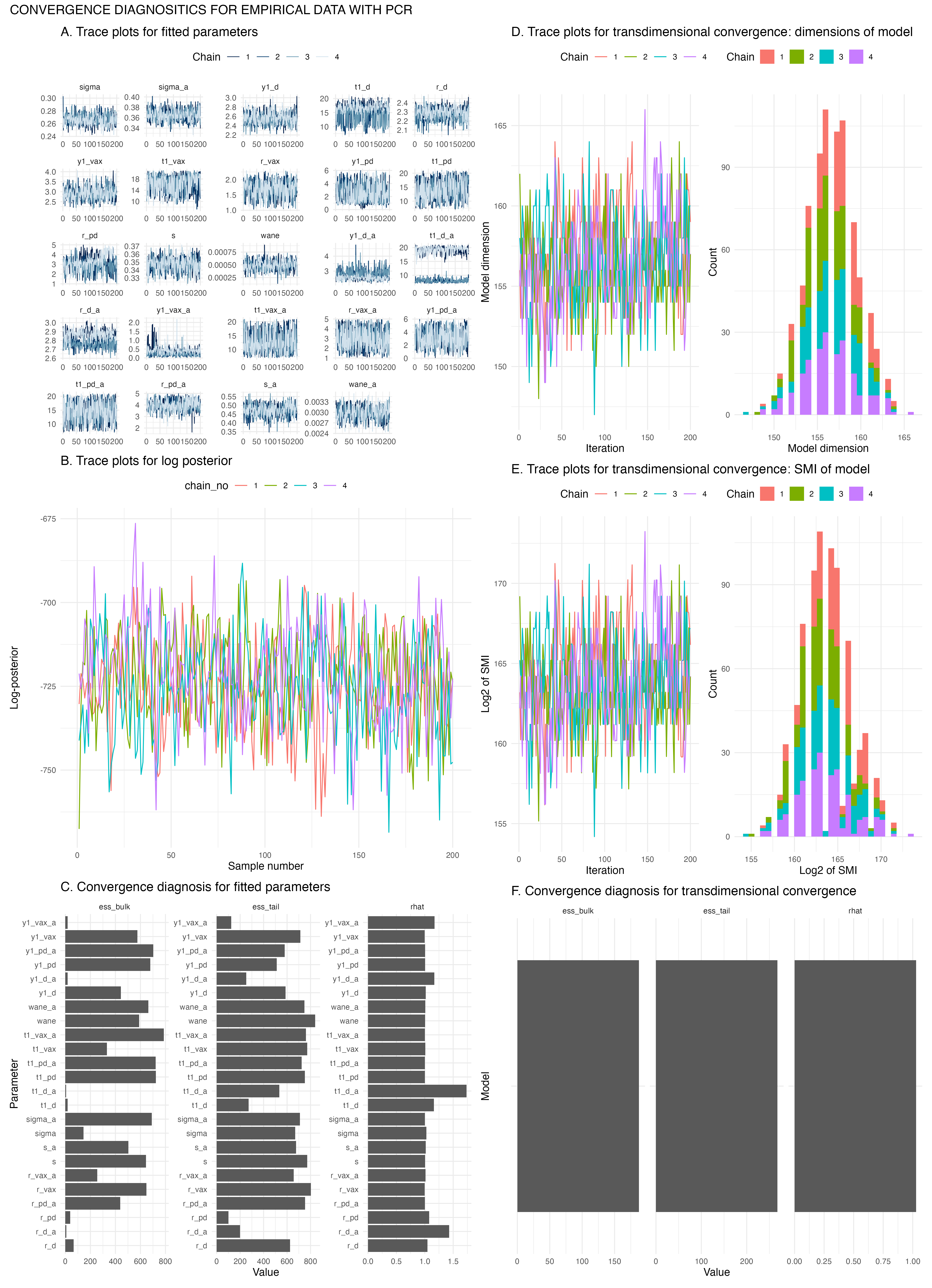

Supplement: Supplement 8 — Figure S7: Convergence diagnostics for empirical data with PCR information (A) Trace plots for fitted parameters (sigma, wane, a, b, c) across four Markov chains, illustrating the mixing and convergence of the parameters. (B) Trace plots for the log posterior across the four chains, showing the variability and stabilization of the log posterior over iterations. (C) Convergence diagnostics for fitted parameters, including effective sample size (ess_bulk, ess_tail) and Rhat, which assess the adequacy of sampling and convergence for each parameter. (D) Trace plots for transdimensional convergence of the model dimension, with histogram counts of model dimensions sampled across the chains. (E) Trace plots for transdimensional convergence for the SMI (Structural Model Index) and histogram counts of the log-transformed SMI values across chains. (F) Convergence diagnostics for transdimensional parameters, including effective sample size (ess_bulk, ess_tail) and Rhat, summarizing the adequacy of sampling and convergence for the transdimensional space. [file media-8.jpg]

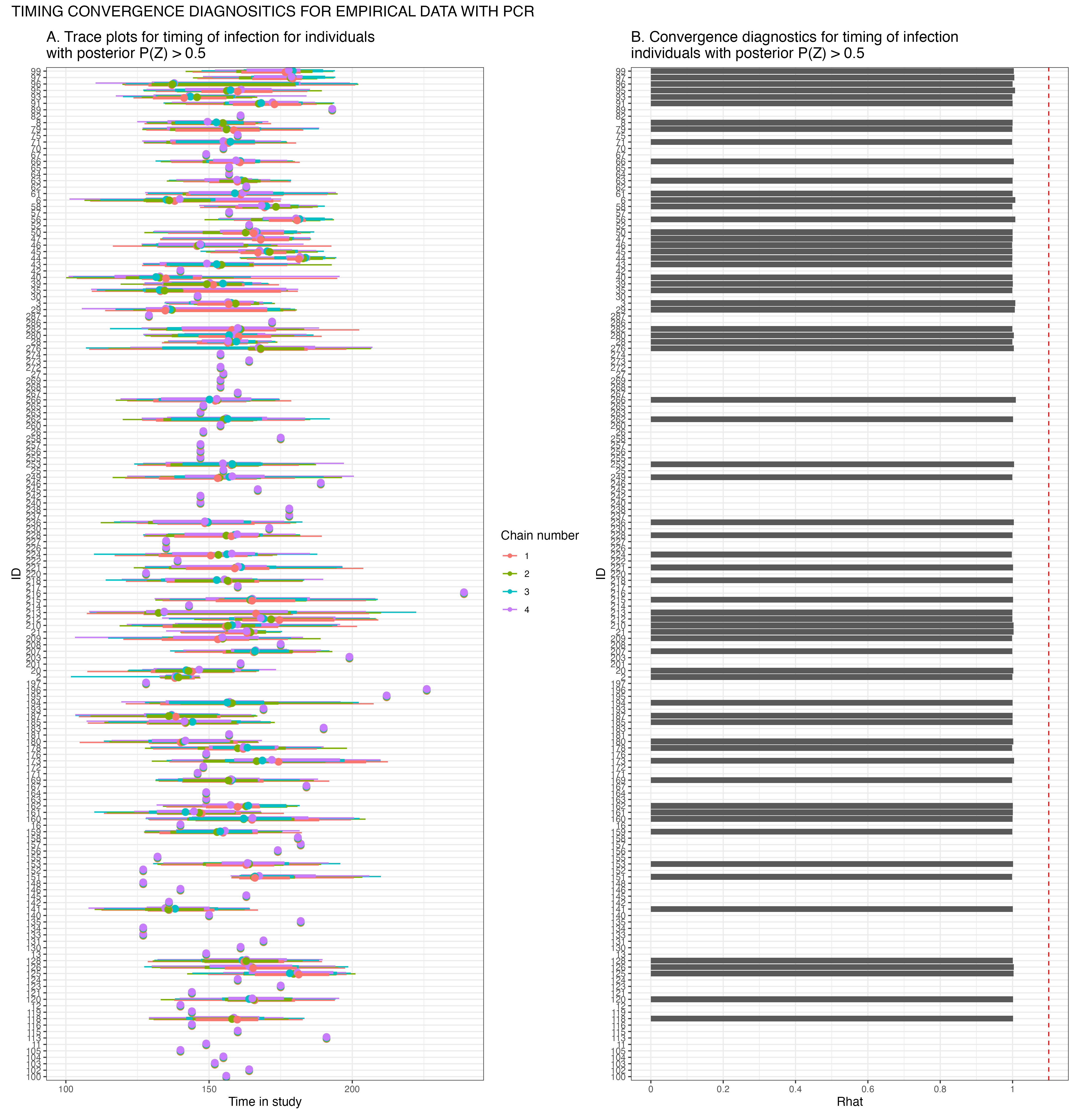

Supplement: Supplement 9 — Figure S8 Convergence diagnostics for empirical data with PCR. (A) Trace plots for the timing of infection for individuals with posterior P(Z) > 0.5 display estimates across four Markov chains. Each point and its uncertainty interval reflect the sampled infection timing for each individual over iterations. (B) Convergence diagnostics for the timing of infection for individuals with posterior P(Z)>0.5, showing Rhat values for each individual. The red dashed line indicates the threshold for Rhat=1.1, which marks convergence. [file media-9.jpg]

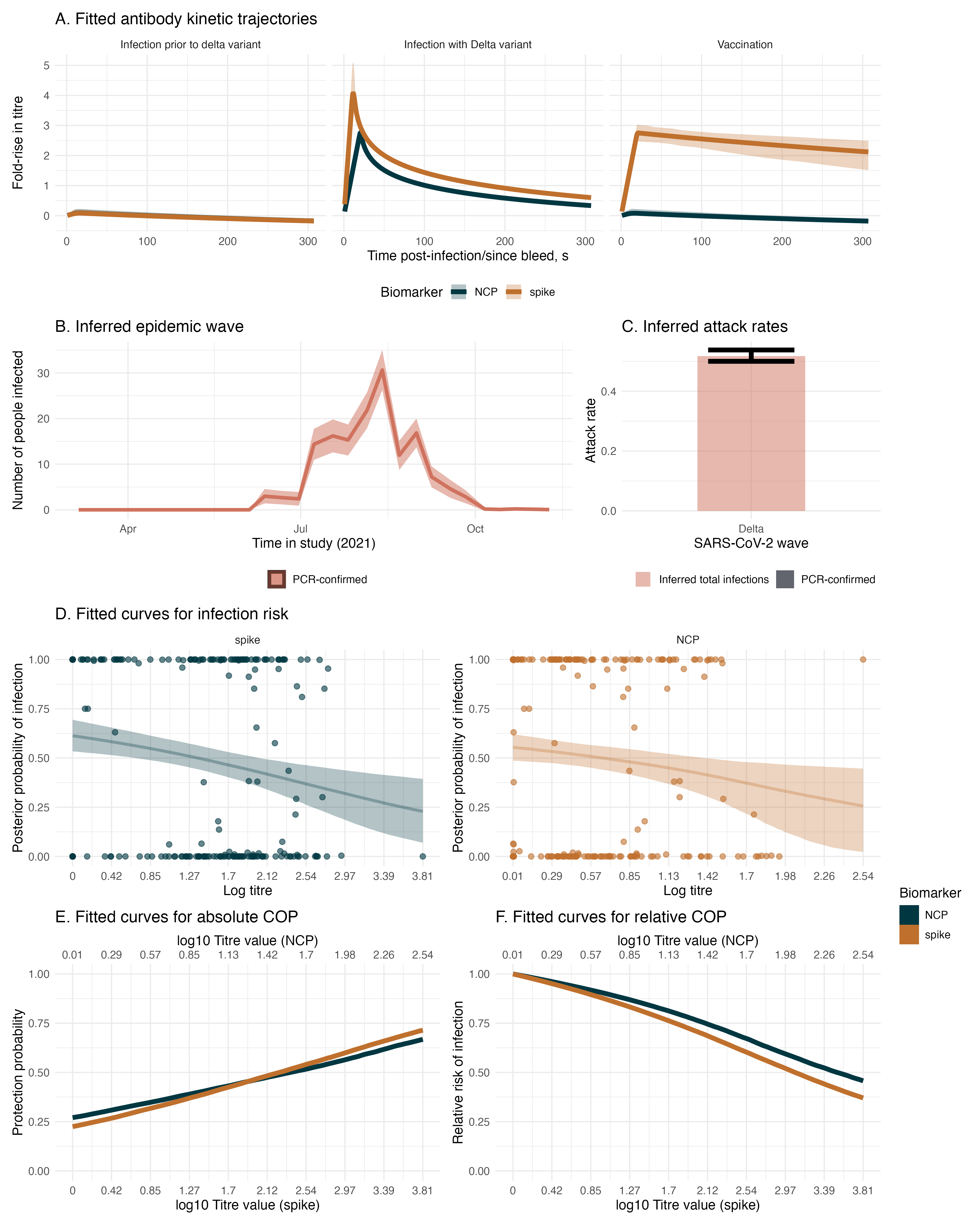

Supplement: Supplement 10 — Figure S9: Antibody kinetics, recovery of infection timings, and correlates of protection from empirical data without PCR information. (A) Fitted antibody kinetic trajectories for individuals with infection prior to the Delta variant (left), infection during the Delta variant wave (center), and following vaccination (right), showing fold-rise in antibody titre over time for NCP (blue) and spike (orange) biomarkers. Shaded regions represent 95% credible intervals. (B) Inferred epidemic wave during the Delta wave, illustrating the temporal distribution of PCR-confirmed cases (black bars) and total inferred infections (pink shaded curve), with shaded uncertainty. (C) The estimated attack rate during the Delta wave, partitioned into PCR-confirmed cases (dark grey) and total inferred infections (pink). Error bars indicate 95% credible intervals. (D) The fitted logistic curve to the infection risk, showing the posterior probability of infection as a function of antibody titre at infection for NCP (blue) and spike (orange) biomarkers, with shaded regions representing 95% credible intervals. (E) The absolute COP from the fitted infection risk curve and (F) shows the relative COP for the same biomarkers. [file media-10.jpg]
